# Effects of Experimentally Induced Lower Limb Muscle Fatigue on Healthy Adults’ Gait: A Systematic Review

**DOI:** 10.3390/bioengineering12030225

**Published:** 2025-02-22

**Authors:** Liangsen Wang, Wenyue Ma, Wenfei Zhu, Lin Zhai, Yuliang Sun

**Affiliations:** School of Physical Education, Shaanxi Normal University, Xi’an 710119, China; wlsen13@snnu.edu.cn (L.W.); mawnyue@snnu.edu.cn (W.M.); wzhu@snnu.edu.cn (W.Z.)

**Keywords:** muscle fatigue, gait, biomechanical

## Abstract

Lower limb fatigue reduces muscle strength, alters joint biomechanics, affects gait, and increases injury risk. In addition, it is of great clinical significance to explore local muscle fatigue or weakness caused by fatigue to understand its compensatory effect on the ipsilateral or contralateral joints. We systematically searched multiple databases, including five databases, using key terms such as “Muscle Fatigue” and “Gait”. Only studies that experimentally induced fatigue through sustained muscle activities in healthy adults were included. This review examined 11 studies exploring the effects of lower limb muscle fatigue on gait and lower limb biomechanics. The findings indicated that muscle fatigue significantly influenced spatiotemporal parameters, joint angles, and moments. Most studies that were reviewed reported an increase in step width and a decrease in knee joint moments following fatigue. Additionally, muscle activation levels tended to decline. In summary, compensatory mechanisms can lead to new walking strategies, such as increasing step width or enhancing the strength of muscles in adjacent joints. These adjustments impact dynamic balance differently: wider steps may enhance medial–lateral stability, while reduced muscle strength could lead to higher heel contact velocity and longer slip distances. Although these changes might influence dynamic balance, compensatory strategies may help mitigate the overall effect of fall risk. Future studies should use appropriate protocols, such as moderate or severe fatigue interventions with isokinetic dynamometry.

## 1. Introduction

According to the US Health and Nutrition Examination Survey, 14.3% of men and 20.4% of women report feeling fatigued [1]. Fatigue is a state of exhaustion caused by the depletion of physical and mental energy, often accompanied by a diminished interest in the surrounding world. Prolonged physical exertion can lead to a decline in athletic performance (i.e., performance fatigue) [2,3,4]. Even prolonged low-intensity activities, such as extended fast-paced walking, can result in muscle fatigue. High-intensity, short-duration exercise tasks can also lead to performance fatigue in muscles [2,5]. Sustained muscular effort causes a reduction in strength, thereby affecting the quality of motor behaviour, such as maintaining body posture and gait stability [6,7]. Walking is the most common form of physical activity [8]. Muscle fatigue has been identified as a significant task-related risk factor affecting gait [9]. Muscle fatigue leads to decreased muscle strength and can impair neuromuscular control, resulting in a higher risk of injuries during walking [9,10,11,12]. Lower limb multi-joint muscle fatigue can increase the ankle plantar flexion angle and slip distance (SD) at heel contact [13]. Local quadriceps muscle fatigue can significantly reduce quadriceps strength and knee joint torque during the stance phase while also decreasing the ankle plantar flexion angle and increasing knee flexion at heel contact. Additionally, it manifested an increase in heel contact velocity (HCV) and the required coefficient of friction (RCOF), thereby increasing the risk of falling [14]. Studies have also found that abnormal gait is a significant injury risk factor [15]. Therefore, understanding the effects of lower limb muscle fatigue on gait is crucial for injury prevention and for understanding diseases linked to lower extremity weakness. Researchers often simulate fatigue in laboratory settings to investigate how fatigue impacts gait and to identify potential injury risks. They aim to analyse the biomechanical changes in the lower limbs following muscle or functional task fatigue protocols and to develop strategies to prevent injuries.

To effectively understand and treat neuromuscular diseases of the lower limbs, it is crucial first to understand the normal biomechanics of lower limb movement in healthy individuals, including the interactions between the neuromuscular and musculoskeletal systems [16]. Scholars investigated the compensation mechanisms following local muscle fatigue or weakness induced by muscle fatigue, including comparisons between the fatigued and non-fatigued regions, as well as between the fatigued and non-fatigued sides. [16,17]. Fatigue can be categorised into peripheral and central fatigue, with both complex and interrelated processes. Research has demonstrated that prolonged physical or mental exertion can lead to decreased performance and reduced ability to allocate cognitive resources for task execution, resulting in peripheral and central fatigue [2,5,18]. Extended periods of sustained attention or cognitive labour can slow cognitive processes, causing central fatigue (neural fatigue). This may indirectly impair top-down cognitive control and motor task execution, even without noticeable peripheral fatigue (muscle fatigue) [19,20]. While central fatigue is important, many studies focus on peripheral fatigue to investigate its impact on other muscles and joints following muscle fatigue. For example, experimentally induced fatigue is a commonly used intervention method [21]. Experimentally induced fatigue is a feasible and versatile model, enabling researchers to select protocols that are flexibly tailored to specific research needs [21]. Unlike activities of daily living (ADL) or sport-specific protocols, experimentally induced fatigue is simpler and more controlled. It is typically achieved through isometric contractions, dynamic exercises, or repetitive tasks, allowing for reproducible investigation of the effects of fatigue. Many studies have revealed that fatigue of single-joint muscle groups in the lower limb affects the biomechanical characteristics of other joints [22,23,24]. Local muscle fatigue of a single joint inevitably affects the biomechanics of adjacent joints, leading to compensatory phenomena between adjacent joints [16]. Fatigue of the plantar flexor muscles increased the knee flexion angle during the loading phase while decreasing both the knee flexion moment and the ankle plantar flexion moment, with these changes typically occurring at the toe-off [16]. Using functional tasks to induce fatigue in the quadriceps and triceps surae muscles, researchers found changes in patterns of muscle exertion. After the fatigue of the plantar flexor and dorsi flexor muscles, the peak torques around the ankle joint decreased, while the peak torques around the knee and hip joints significantly increased. In kinetic studies, the primary exertion muscles shifted from the fatigued to the non-fatigued areas [25].

Therefore, researching gait after lower limb muscle fatigue is essential, whether for preventing fall injuries or understanding compensatory mechanisms, including comparisons between fatigued and non-fatigued regions and sides. Although many studies have been published on the effects of laboratory-induced lower limb muscle fatigue on gait, their study designs, fatigue protocols, indicator parameters, and research results still need to be revised. This study aimed to systematically review existing research comparing the effects of experimentally induced muscle fatigue on gait kinematics, kinetics, and muscle activity in healthy adults. A comprehensive review of these adaptations is timely and essential, as it will deepen our understanding of the underlying changes in lower limb joints during walking under fatigued conditions.

## 2. Materials and Methods

This systematic review followed the Preferred Reporting Items for Systematic Reviews and Meta-Analyses (PRISMA) guidelines [26]. It has been registered in the International Prospective Register of Systematic Reviews (PROSPERO) with the identifier CRD42025642424. We provided our PRISMA checklist in the supplementary uploaded files, which covers all the key elements required for reporting systematic reviews.

### 2.1. Search Strategy

Several studies examining the effects of lower limb fatigue on gait were retrieved from various databases, including Web of Science, PubMed, CINAHL, MEDLINE, and Cochrane, using specific search terms based on the study’s Population, Intervention, Comparator, Outcomes, and Study Design (PICOS) framework. The first search included studies published up to September 2024; a second search was conducted in January 2025. We presented our search strategy and the PICOS framework for the systematic review study design in Table 1 and Table 2. After the search, all studies were imported into EndNote X9 (Clarivate Analytics, Philadelphia, PA, USA), and duplicate studies were removed [27].

### 2.2. Inclusion and Exclusion Criteria

Studies were considered for inclusion if they met the following criteria: (1) any study design, excluding animal experiments and any type of review; (2) participants aged 18 to 59 years; (3) fatigue protocols using isokinetic dynamometry training and other experimental methods which can non-invasively induce muscle fatigue in the lower limbs; (4) the research field within sports sciences; (5) assessment measures include kinematics, dynamics, electromyographic indicators, and balance metrics. Studies were excluded if they met any of the following criteria: (1) studies with participants outside the age range of 18 to 59 years; (2) animal studies or any type of review (systematic reviews, meta-analyses, etc.); (3) studies where invasive methods or non-laboratory fatigue protocols induced muscle fatigue; (4) studies that did not focus on the field of sports science (e.g., studies outside of physical exercise, rehabilitation, or sports-related contexts); and (5) studies that did not include kinematic, kinetic, electromyographic, or balance assessments as outcome measures.

### 2.3. Studies Selection

The study selection process included (1) selecting studies based on title screening, (2) selecting studies by reviewing abstracts, and (3) selecting studies through full-text reading, primarily focusing on the methods section while considering the established inclusion criteria. Potentially eligible studies were also identified by reviewing reference lists. Three independent reviewers extracted data (L.Z., W.Z., W.M.), and any discrepancies were discussed among the reviewers to reach a consensus.

### 2.4. Data Extraction

Table 3 and Table 4 documented the study data information, including (1) authors, (2) publication year, (3) participant characteristics, (4) study type, (5) fatigue protocols, (6) kinematic dynamics data, spatiotemporal gait characteristics and others. Three independent reviewers extracted the data (L.Z., W.Z., W.M.), and any discrepancies were discussed among the reviewers to reach a consensus.

### 2.5. Risk of Bias and Study Quality Assessment

Two authors (L.Z. and W.Z.) independently assessed the methodological quality of the included studies using a quality appraisal tool that evaluates internal validity, external validity, and generalizability. Due to the observational cross-sectional design of the included studies, many conventional tools for assessing the quality of randomised controlled trials were not applicable. Therefore, the Downs and Black checklist was employed to evaluate the methodological quality of the included studies [28]. This checklist is suitable for all quantitative study designs and comprises five subdomains: reporting quality (items 1–8), external validity (items 9–11), internal validity (items 12–15), and statistical power (item 16). Each item was scored as 1 point for “Yes” and 0 points for “No” or “Unable to determine” [29,30]. The total quality score for each study was calculated out of a maximum of 16 points. Any discrepancies between the two authors were resolved through discussion, and if a consensus could not be reached, a third author (W.M.) was consulted for the final inclusion decision (Table 5).

**Table 3:** Characteristics of the studies: isokinetic dynamometer.

	Michael A. Hunt., 2017 [16]	Fui Ling Lew., 2014 [13]	Heather S. Longpré., 2012 [22]	G. H. Murdock., 2011 [23]	Urs Granacher., 2010a [31]	Urs Granacher., 2010b [24]	Prakriti Parijat., 2008 [14]
Participants	18	60	20	20	16	14	16
Male/Female (N)	9/9	35/25	0/20	10/10	8/8	14/0	10/6
Age (Mean ± SD in Yrs)	25.2	23.1 ± 1.7 ^a^ 24.2 ± 3.0 ^b^ 23.5 ± 1.4 ^c^	18–30	19–35	24.3 ± 1.4	27 ± 3.1	24.7 ± 3.9
Gait studies	Level Gait	Level Gait	Level Gait	Level Gait	Level Gait	Level Gait	Level Gait
Isokinetic dynamometer	Biodex Medical Systems	Biodex Medical Systems	Biodex Medical Systems	Cybex International Inc.	Cybex International Inc.	Cybex International Inc.	Biodex Medical Systems
Fatigue of muscles	Ankle	Ankle, knee, and hip	Knee	Knee	Knee	Ankle	Knee
Muscle contraction	Ankle plantar flexion	Hip and knee extension, ankle plantar flexion	Knee flexion and extension	Knee extension	Knee extension	Ankle plantar and dorsiflexion	Knee flexion and extension
Type of muscle contraction	Isokinetic contraction	Isotonic contraction	Isotonic contraction	Isokinetic contraction	Isokinetic contraction	Isokinetic contraction	Isokinetic contraction
Fatigue regimen	Plantar flexion contractions at 180°/s	Ten lifts/min 60% MVC	50% MVC	Knee extension at 90°/s	Knee flexion and extension at 60°/s	Ankle plantar and dorsiflexion at 60°/s	Knee flexion and extension at 60°/s
Contraction type	Concentric			Concentric	Concentric–concentric	Concentric–concentric	Concentric–concentric
Unilateral/bilateral	Unilateral	Bilateral	Unilateral	Unilateral	Bilateral	Unilateral	Bilateral
Fatigue evaluation	<60% MVC	<70% MVC three times in a row	<75% MVC	<50% MVC	<50% MVC	<50% MVC	<60% MVC

^a^, No fatigue group (13 males and seven females); ^b^, Lower-limb fatigue group (10 males and 10 females); ^c^, Upper-limb fatigue group (12 males and eight females); Maximum Voluntary Contraction (MVC).

**Table 4:** Characteristics of the Studies: Cycle Ergometer, Medical Treadmill, and Sit-to-Stand Task.

	Dennis Hamacher., 2016 [32]	F.A. Barbieri., 2014 [25]	Fabio Augusto Barbieri., 2013 [33]	Xingda Qu., 2011 [34]
Participants	10	10	20	12
Male/Female (N)	NE	5/5	20/0	12/0
Age (Mean ± SD in Yrs)	23.0 ± 4.8	27.6 ± 2.7	24.7 ± 2.8	26.6 ± 2.9
Gait studies	Level Gait	Step gait	Level Gait	Level Gait
Fatigue equipment	Cycle ergometer	Sit-to-stand task	Sit-to-stand task	Medical treadmill
Fatigue of muscles	Lower limbs	Quadriceps and triceps calves	Lower limbs	Lower limbs
Fatigue regimen	Starting workload: 50 watts; Incremental workload: 25 watts; Stage length: 3 min; Additional charge: 3 min by 25 watts; Cadence: 70–80 rpm;	A standard chair (40 cm high, 40 cm wide, 35 cm deep; frequency of 0.5 Hz	A standard chair (43 cm high, 41 cm wide, 42 cm deep; 30 beats/min)	2 and 10 min; 8 mph
Fatigue evaluation	RPE: 18	Below 0.5 Hz or after 30 min	Below 30 beats/min or after 30 min	RPE: 17
Model	Xrcise Cycle Med, Cardiowise^®^, Pirmasens, Germany	NE	NE	Biodex RTM 600, Shirley, NY, USA

Borg’s rating of perceived exertion (RPE), and NE: The study does not carefully explain.

**Table 5:** Summary of quality appraisal for individual studies according to the Downs and Black checklist.

First Author and Years	Michael A. Hunt., 2017 [16]	Fui Ling Lew., 2014 [13]	Heather S. Longpré., 2012 [22]	G. H. Murdock., 2011 [23]	Urs Granacher., 2010a [31]	Urs Granacher., 2010b [24]	Prakriti Parijat., 2008 [14]	Dennis Hamacher., 2016 [32]	F.A. Barbieri., 2014 [25]	Fabio Augusto Barbieri., 2013 [33]	Xingda Qu., 2011 [34]
Reporting											
1. Is the hypothesis/aim objective of the study clearly described?	Y	Y	Y	Y	Y	Y	Y	Y	Y	Y	Y
2. Are the main outcomes to be measured clearly described in the Introduction or Methods sections?	Y	Y	Y	Y	Y	Y	U	U	Y	U	Y
3. Are the characteristics of the patients included in the study clearly described?	Y	Y	Y	Y	Y	Y	Y	Y	Y	Y	Y
4. Are the interventions of interest clearly described?	Y	Y	Y	Y	Y	Y	Y	Y	Y	Y	Y
5. Are the distributions of principal confounders in each group of subjects to be compared clearly described?	Y	Y	N	Y	Y	Y	Y	N	Y	Y	Y
6. Are the main findings of the study clearly described?	Y	Y	Y	Y	Y	Y	Y	Y	Y	Y	Y
7. Does the study provide estimates of the random variability in the data for the main outcomes?	Y	Y	Y	Y	Y	Y	Y	Y	Y	Y	Y
8. Have actual probability values been reported for the main outcomes except where the probability value is less than 0.001?	Y	Y	Y	N	Y	Y	Y	Y	Y	Y	Y
External Validity											
9. Were the subjects asked to participate in the study representative of the entire population from which they were recruited?	Y	Y	N	Y	Y	N	Y	N	Y	N	N
10. Were those subjects who were prepared to participate representative of the entire population from which they were recruited?	Y	Y	N	Y	Y	N	Y	N	Y	N	N
11. Were the staff, places, and facilities where the patients were treated representative of the treatment the majority of patients receive?	Y	Y	Y	Y	Y	Y	Y	Y	Y	Y	Y
Internal Validity											
12. Were the statistical tests used to assess the main outcomes appropriate?	Y	Y	Y	Y	Y	Y	Y	Y	Y	Y	Y
13. Were the main outcome measures used accurate (valid and reliable)?	Y	Y	Y	Y	Y	Y	U	Y	Y	Y	Y
Internal Validity (Confounding, Selection Bias)											
14. Were the patients in different intervention groups (trials and cohort studies), or were the cases and controls (case–control studies) recruited from the same population?	Y	Y	Y	Y	Y	Y	Y	Y	Y	Y	Y
15. Was there adequate adjustment for confounding in the analyses from which the main findings were drawn?	U	U	U	U	U	U	U	U	U	U	U
Power											
16. Did the study have sufficient power to detect a clinically important effect where the probability value for a difference being due to chance is less than 5%?	U	U	Y	U	U	U	U	N	Y	N	Y
Total Score											
Score/16	14	14	12	13	14	12	12	10	15	11	13

Abbreviations: Y, yes; N, no; U, unable to decide.

### 2.6. Data Analysis

Due to the heterogeneity of the results, the lack of consistent findings, and the limited number of eligible studies, we could not conduct a meta-analysis. The variables involved in the studies were described through qualitative or quantitative analyses, along with a narrative of the study outcomes. We conducted a descriptive statistical survey of the articles using factors such as fatigue Protocol.

## 3. Results

### 3.1. Studies Inclusion and Characteristics

**Figure 1:**
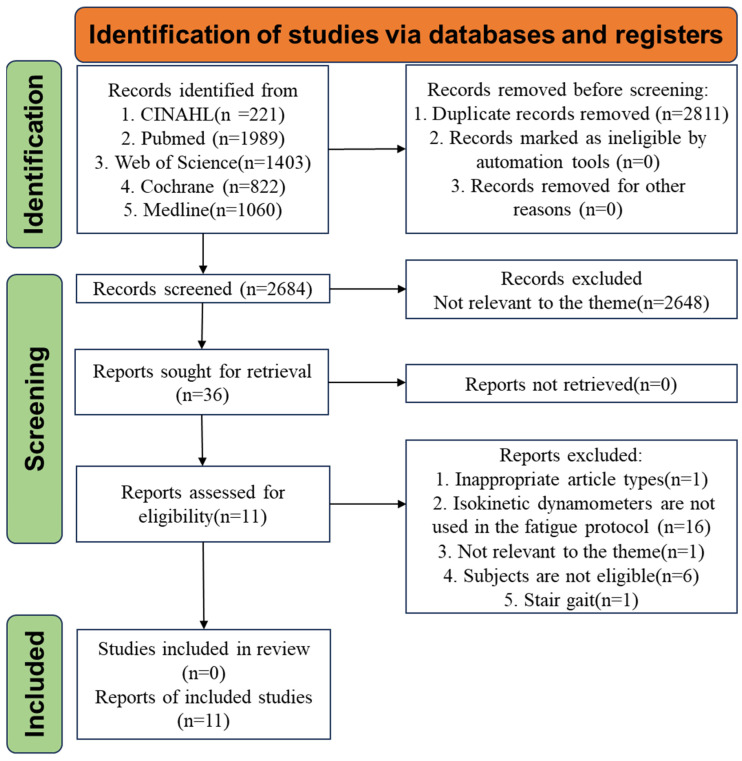
Studies selection process and inclusion process.

Database searches yielded 2684 studies, of which 2648 were excluded after reading the titles and abstracts, and 36 were considered potentially eligible. Additionally, 25 studies were excluded after reading the full texts. Eleven studies met the inclusion criteria and were included in the review. The entire eligibility assessment process is illustrated in Figure 1. The eleven studies (n = 216, male = 123, female = 83, NE = 10) were published between 2008 and 2017.

### 3.2. Quality Assessment

The quality scores of the studies ranged from 10 to 11 out of 16 for two studies, from 12 to 13 for five studies, and exceeded 14 for 4. All included studies provided accurate main outcomes (valid and reliable). The most notable issues involved external validity, selection bias, and statistical power, for example, whether the participants and study settings were representative of the general population, whether the primary findings adequately accounted for confounding factors, and the unclear clinical significance of many studies (Table 3).

### 3.3. Fatigue Protocol

Increasing research explores the effects of lower limb fatigue on gait and the biomechanics of lower limb joints. Laboratory-induced lower limb fatigue protocols should be quick and convenient. Based on the laboratory conditions, selecting a safe, efficient protocol that induces fatigue and leads to relatively slow recovery is essential. This is a pressing issue that must be addressed. Although all studies effectively induced fatigue in participants, the protocols used varied significantly.

These studies used different protocols to induce lower limb muscle performance decline, including repetitive sit-to-stand transitions [25,33] (n = 2), endurance fatigue [32,34] (n = 2, treadmill and cycling), and isokinetic protocols (n = 8). All studies indicated that a decrease in peak joint torque characterised fatigue, a perceived exertion (RPE) rating of 17 or higher, an inability to continue exercising, or a reduction in movement frequency (Table 4 and Table 5).

#### 3.3.1. Isokinetic Dynamometry

Eight studies used isokinetic dynamometry as their fatigue protocol among the retrieved articles. Isokinetic dynamometry is considered the gold standard method for strength assessment and is fundamental for measuring muscle strength and joint function [35,36]. This device applies resistance at a constant speed to match the participant’s strength, measuring parameters such as joint torque, total muscle work, and power [37]. Isokinetic dynamometry quantitatively evaluates muscle function, and its measurements are safe, objective, and reproducible [38]. All included studies that utilised isokinetic contraction modes selected concentric contraction. Table 3 systematically summarised key factors related to muscle fatigue and performance. This includes the brand of the isokinetic dynamometer used, the specific muscle groups where fatigue occurred, and the type of muscle contraction involved in fatigue. Additionally, we detailed the contraction type, the fatigue regimen used to induce fatigue, whether the protocol involved unilateral or bilateral muscle engagement, and the methods employed for fatigue evaluation. This comprehensive summary provided a clear framework for understanding the dynamic changes in muscle performance under fatiguing conditions.

Studies that used isokinetic dynamometry to induce lower limb fatigue used various contraction methods targeting different joints. They used an isokinetic dynamometer (with a lift simulation attachment) with isotonic contractions to fatigue the multi-joints of both lower limbs. During the training, participants performed push exercises on the lift simulator attachment of the dynamometer, lying on the floor without moving their trunk and upper limbs. Repetitive lower limb push movements were used to induce lower limb fatigue, involving the ankle, knee, and hip joints, with minimal upper limb movement. The load was set at 60% MVC, and the required speed was ten lifts per minute [15,39]. One study used a dynamometer (with a leg extension attachment) with isotonic contractions to fatigue a single knee joint. The knee joint performed repetitive flexion and extension against a load of 50% MVC until the knee joint torque was less than 75% MVC [22]. Others used isokinetic dynamometry with isokinetic training to fatigue the knee joint, all for unilateral fatigue. For knee joint fatigue, the knee extension speed was set at 60°/s or 90°/s, and the flexion at 120°/s [14,23,31]. Since the quadriceps are the primary muscles engaged during walking, these speeds were selected to simulate quadriceps fatigue, making knee extension more strenuous while reducing the difficulty of flexion. Fatigue indicators were assessed when joint torque was less than 50% MVC or less than 60% MVC. Studies have found that a 40% decrease in joint torque was clinically relevant as it falls within the range of moderate to severe muscle fatigue of the knee extensors and flexors [40,41,42]. Two studies used isokinetic dynamometry with isokinetic training to fatigue the plantar flexor and dorsiflexor muscles, all for unilateral fatigue [16,24]. For fatigue of the plantar flexor and dorsiflexor muscles, repetitive dorsiflexion was performed at speeds set at 60°/s or 180°/s, with fatigue indicators being joint torque less than 50% MVC or less than 60% MVC [16,24]. Most fatigue protocols selected a speed range between 60°/s and 90°/s, with only one study choosing a speed of 180°/s. However, this study also reported that four participants did not reach the desired level of fatigue following the intervention [16]. To some extent, it was found that a speed range of 60°/s to 90°/s was more effective. The chosen isokinetic dynamometers in the studies were from Biodex Medical Systems or Cybex International Inc. There was no difference in fatigue outcomes between the devices. It was recommended that equipment that can accommodate bilateral simultaneous activities and multi-joint movements be selected to facilitate various fatigue interventions in the future.

#### 3.3.2. Sit-to-Stand Task

Two articles used the sit-to-stand task fatigue protocol, as the muscles used in standing and sitting correlate highly with those used in walking [25,33]. The chairs used were 40 cm high, 40 cm wide, 35 cm deep, or 43 cm high, 41 cm wide, and 42 cm deep. The action frequency was set with a metronome, completing 30 sit-to-stand cycles per minute. The evaluation criteria were based on the subjective inability to continue the action or failure to maintain the specified action frequency, serving as reliable indicators of fatigue. The action requirements were as follows: Participants were instructed to cross their arms in front of their chest, ensure their knees were fully extended while standing, and then sit down. They repeated this process according to the metronome rhythm until they could no longer perform the task.

#### 3.3.3. Bicycle Ergometer and Treadmill Tasks

One study used a cycle ergometer (Xrcise Cycle Med, Cardiowise^®^, Germany) to fatigue the lower limbs, with the following parameters: starting workload: 50 watts; incremental workload: 25 watts; stage length: 3 min; additional charge: 3 min by 25 watts; cadence: 70–80 rpm [32]. The evaluation criterion was reaching an RPE of 18, indicating fatigue. One study used a medical treadmill (Biodex RTM 600, Shirley, NY, USA) to fatigue the lower limbs, with the following parameters: duration: 2 and 10 min; speed: 8 mph [34]. The evaluation criterion reached an RPE of 17, indicating fatigue; however, it is important to note that central fatigue could influence the rating, as RPE is subjective and may reflect both peripheral and central components of fatigue.

In summary, the isokinetic dynamometer may undoubtedly be the best method regarding laboratory fatigue protocols. It is quick, safe, and provides real-time feedback. Although the treadmill requires relatively little time, about 10 minutes, it induced more overall fatigue, including cardiopulmonary endurance fatigue. The sit-to-stand task uses muscles similar to those used in walking but is slower, generally requiring 20 min or more, which is time-consuming in experiments. The isokinetic dynamometer can precisely fatigue a single joint or the entire lower limb and adjust the fatigue model to meet most requirements. The most suitable speed for lower limb fatigue was 60°/s, and the fatigue indicator can be set as joint torque less than 50% MVC.

### 3.4. Fatigue of Muscles

In daily activities and occupational environments, bilateral fatigue is more common than unilateral fatigue, and multi-joint movements are more frequent than single-joint movements. The movement of one joint often depends on the position of adjacent joints [43]. Therefore, studying bilateral fatigue can effectively reveal the impact of fatigue on gait. Applying local muscle fatigue protocols to a single lower limb joint is also significant for exploring the biomechanical characteristics between various lower limb joints. Among the retrieved articles, seven studies used bilateral fatigue strategies ((isokinetic dynamometer = 3 [13,14,31], endurance fatigue (cycle ergometer, medical treadmill, and sit-to-stand task) = 4 [25,32,33,34])), and four studies used unilateral fatigue strategies (isokinetic dynamometer = 4 [16,22,23,24]). There were five articles on multi-joint fatigue (isokinetic dynamometer =1 [13], endurance fatigue = 4 [25,32,33,34]) and six on single-joint fatigue (ankle = 2 [17,24], knee = 4 [16,22,23,31]).

### 3.5. Biomechanical Methods and Indicators

The assessment of gait using the synchronisation of kinematic kinetic and surface electromyography (sEMG) data is commonly used in biomechanics research. Our systematic review found that ten articles used kinematic methods, eight used kinetic methods, four used EMG, and one used accelerometers. Three studies used kinematic, kinetic, and sEMG methods together (Table 6 and Table 7). 

#### 3.5.1. Kinematic and Kinetic

For gait analysis, capture systems included Qualisys, Vicon, and Kistler force plates. Most studies instructed participants to walk over a 10 m level-ground walkway that contained one or more floor-embedded force plates. Kinematic and kinetic studies primarily used conventional motion capture systems and three-dimensional force plates synchronised with sEMG signal collection.

One study that used isokinetic dynamometers to induce unilateral fatigue of the plantar flexor and dorsiflexor muscles found that fatigue reduced both the knee flexion moment and the ankle plantar flexion moment, primarily occurring at the toe-off. In contrast, the knee joint angle increased during the loading phase [16]. Conversely, when bilateral lower limb fatigue was induced using isokinetic dynamometers, researchers observed greater ankle plantar flexion at heel contact compared to the no-fatigue condition [13]. However, findings on ankle joint angles remain controversial. Some studies reported minimal changes in the knee flexion angle, while others noted that muscle fatigue of the knee extensors and flexors increased both plantar flexion and knee flexion angles at heel contact. Additionally, the knee extension moment significantly decreased during the stance phase following fatigue [14]. Regarding muscle fatigue of the knee extensor, studies have shown that fatigue significantly increased the knee adduction angle, particularly during the swing phase. In contrast, the knee flexion moment during the early stance phase was significantly reduced post-fatigue [23]. Furthermore, fatigue led to a decrease in the peak knee extension moment, observed between 40% and 50% of the gait cycle, coinciding with the phase when the heel leaves the ground in preparation for push-off [22]. This discrepancy may arise from differences in fatigue protocols, such as variations between isotonic and isokinetic training or in the speed selection for isokinetic training.

#### 3.5.2. sEMG

Regarding the use of sEMG, we observed some differences in the selection of lower limb muscles. Hunt et al. [16] selected seven lower limb muscles for sEMG: vastus lateralis (VL), vastus medialis (VM), rectus femoris (RF), lateral hamstring (LH), medial hamstring (MH), and lateral (LG) and medial (MG) gastrocnemius muscles. This selection aimed to investigate the biomechanics of the ankle and knee during normal walking after the fatigue of the plantar flexor muscles. Heather et al. [22] selected three muscles for sEMG: rectus femoris, vastus lateralis, and lateral hamstring. This selection aimed to investigate the biomechanical changes in the knee joint following lower limb fatigue in healthy young women. Rectus femoris is the antagonistic muscle that connects the knee and hip joints. As detected by EMG, changes in the activation of the vastus lateralis were associated with significant alterations in knee kinematics during walking or stair climbing. Therefore, this muscle was chosen as a potential explanatory variable for the mechanical changes in the knee joint during gait [44,45]. Murdock et al. [23] placed sEMG on vastus lateralis, vastus medialis, rectus femoris, lateral hamstring, medial hamstring, and lateral and medial gastrocnemius to explore the impact of a high-intensity quadriceps fatigue training protocol on knee joint mechanics and muscle activation during gait in young adults. Granacher et al. [24] placed sEMG on the tibialis anterior, peroneal muscles, and vastus medialis to investigate the effects of fatigue of the plantar flexor and dorsiflexor muscles on gait activities in young men. Although the tibialis anterior plays a crucial role in normal gait, many studies did not apply sEMG to the tibialis anterior. This may be because some past research studies focused on muscles that generate greater force during gait, such as the quadriceps or gastrocnemius. However, the contribution of the tibialis anterior to gait stability and efficiency should not be overlooked, as it works synergistically with other key muscles (e.g., rectus femoris and medial gastrocnemius) to ensure proper biomechanical function [46], particularly during the mid-swing phase where it was crucial for foot clearance [47], and during the loading response (rectus femoris), where it controls ankle plantar flexion as the foot transitions from heel contact to foot flat.

In summary, sEMG attachment site selection varied based on the primary research focus. For a more comprehensive gait study involving the lower limbs, the EMG attachment sites should include the vastus lateralis, vastus medialis, rectus femoris, lateral hamstring, medial hamstring, and lateral and medial gastrocnemius muscles, covering both the anterior and posterior muscle groups of the thigh and the calf. The tibialis anterior should also be included in studies focusing on the ankle joint. The sEMG studies revealed subtle changes in muscle activation patterns. One study noted that the sEMG signals from the rectus femoris and the posterior thigh muscles remained unchanged, whereas another study observed that the median power frequency (MPF) of the rectus femoris, vastus medialis, and vastus lateralis muscles significantly decreased after fatigue [22,23].

#### 3.5.3. Spatiotemporal Parameters

Research on spatiotemporal gait parameters yielded diverse findings. Some studies reported unchanged gait velocity but increased stride frequency [16] or stable gait velocity and stride length [22], indicating consistent gait performance. Others observed reduced gait velocity and stride length [14,31], while stride length variability remained unchanged [31]. Additionally, several studies noted increased step width alongside unchanged stride length and step duration [25,34], suggesting enhanced medial and lateral stability. In contrast, one study reported increased gait velocity, step width, and stride length with decreased step duration [33]. These contradictions may be due to differences in the fatigue protocols.

#### 3.5.4. Dynamic Balance

The selection of relevant indicators for assessing movements’ stability, efficiency, and safety also varied in the papers that were reviewed. Several metrics to assess dynamic stability during gait were identified in the papers we reviewed: (1) heel contact velocity (HCV), which is the speed at which the heel contacts the ground during the gait cycle and serves as a crucial parameter for evaluating gait stability and slip risk [14]; (2) slip distance (SD), which measures the distance slipped when the foot contacts the ground due to insufficient friction and is a vital indicator for assessing slip risk [13]; (3) the required coefficient of friction (RCOF), representing the minimum friction necessary to maintain gait stability and prevent slipping [13]; and (4) lateral dynamic stability (LDS), which denotes an individual’s ability to maintain a stable gait in response to minor perturbations [32]. However, there did not appear to be a consensus about which metric or combination of metrics was most useful to assess dynamic stability.

Two studies yielded different results. One study found that after fatigue, the heel contact velocity (HCV) significantly decreased, while slip distance (SD) significantly increased; however, the required coefficient of friction (RCOF) did not show significant changes [13]. On the other hand, another study observed that after fatigue, both the heel contact velocity (HCV) and the required coefficient of friction (RCOF) significantly increased [14]. This discrepancy may be due to differences in the fatigue protocols used. Additionally, other research has found that after fatigue, local dynamic stability (LDS) during walking significantly increased [32].

## 4. Discussion

**Figure 2:**
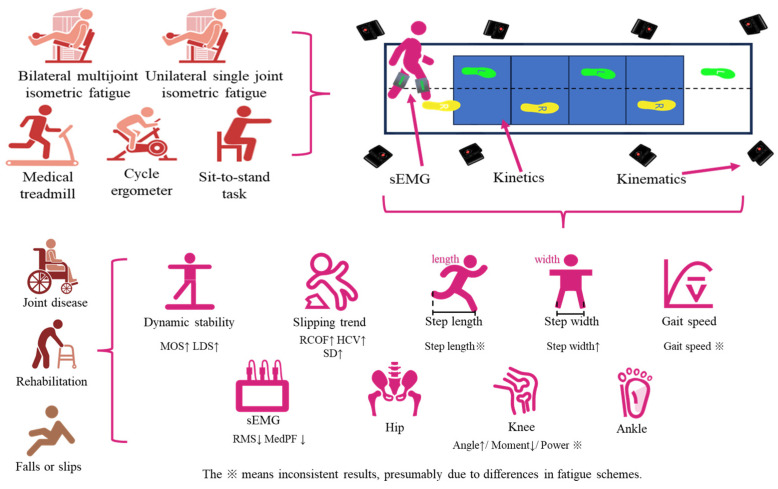
Summary diagram of research content.

Fatigue can affect gait characteristics, increasing the risk of instability and falls. Concurrently, muscle injuries and patients with muscle weakness due to neuromuscular diseases may exhibit similar gait patterns. We have observed instances of compensatory effects on the ipsilateral or contralateral joints. Understanding these impacts is of paramount importance. While most studies focused on central or proximal joint muscle fatigue, fewer studies have systematically examined the impact of lower limb muscle fatigue on gait. Given the importance of the lower limbs in maintaining stability, it is essential to investigate how muscle fatigue affects gait kinematics, dynamics, stability, and muscle activity. This study systematically reviewed the effects of lower limb muscle fatigue on gait in healthy adults. Fatigue reduced joint torque, angles, and muscle activity in the fatigued joints, while non-fatigued joints showed increased torque and muscle activity. These changes occurred in the knee and ankle joints, though there was limited research on the hip joint. Almost all studies found that fatigue leads to reduced gait velocity (GV) and stride length (SL), increased stride width (SW), and more significant stride length variability (SLV). However, there were exceptions, likely due to differences in fatigue protocols, suggesting the need for further validation in future studies (Figure 2).

### 4.1. The Impact of Fatigue Protocol

Research has shown that all muscle fatigue protocols discussed in this study are effective, significantly decreasing lower limb strength. However, there were considerable differences between the fatigue protocols, including (1) using isokinetic dynamometers for repetitive muscle contractions of the lower limb extensors or flexors, (2) sit-to-stand tasks at fast or fixed speeds, and (3) endurance fatigue tasks performed on a treadmill or cycle ergometer. These variations in fatigue induction methods were one reason for the inconsistencies in gait effects reported in studies [21]. We must be cautious regarding these subtle changes, as many studies report no changes in gait metrics following various muscle fatigue protocols, resulting in inconsistent data outcomes. Long-term fatigue was challenging to assess; participants often began gait testing within 5 min after completing the fatigue protocol to minimise recovery effects [23]. One study suggested a 10-min timeframe [14]. Although some parameters may show significant recovery, many indicators still reflect kinematic and kinetics change [14]. These effects might be even more pronounced if the duration of fatigue were longer. This presents a challenge, as high-intensity fatigue effects may not accurately represent the low-level activation fatigue observed in daily activities. In reality, isolated joint muscle fatigue in the lower limbs is rare; activities usually involve prolonged, repetitive variations, as seen in activities of daily living (ADL). When applying a relatively constant load to the knee extensors and flexors, researchers should include sudden changes in joint motion speed to simulate walking fatigue accurately [48]. However, walking and similar activities were not strictly isokinetic or isotonic tasks, as contraction speeds and internal and external loads vary throughout the cyclic motion [22]. Studies have found that young individuals exhibit different activation characteristics of the vastus lateralis, vastus medialis, and rectus femoris under various conditions following isotonic and isokinetic fatigue interventions [45,49]. As a step toward a more functionally relevant representation of these ADLs, an isotonic fatigue protocol was used where the load was held constant while undergoing variations in angular velocity, applied to the flexion and extension of lower limb joints [22]. Therefore, selecting a fatigue protocol was crucial, as different protocols simulate varying scenarios and should be chosen based on the research objectives. To simulate daily activities, we should employ activities of daily living. In contrast, we should utilise an isokinetic dynamometer to investigate localised muscle weakness or muscle injury to perform isokinetic or isotonic contractions.

### 4.2. Effect of Fatigue on Gait

Research has found that even when participants exert maximum effort during approximately 50 knee extensions or ankle plantar flexions [16,23], resulting in a 50% reduction in MVC, some individuals show no changes in gait spatial–temporal parameters. One possibility for this lack of significant gait change is that the torque and force demands during gait remain below the joint torque and force that fatigued muscles can produce [16]. Gait requires relatively low muscle activation levels; during the weight-bearing phase of the gait cycle, rectus femoris activity in young, healthy individuals was typically around 20% of its MVC [50]. Adults with higher lower limb strength retain enough strength after fatigue protocols to engage in submaximal activities like walking without risking potential knee biomechanics injury [22]. Further evidence suggests that participants can compensate for fatigue by activating unaffected muscles during tasks. This represents a walking strategy that helps to maintain a functional gait despite fatigue. Specifically, fatigue in the triceps surae was associated with increased muscle activity in the biceps femoris during standing and in the rectus femoris during the swing phase [25,51]. After fatigue of the plantar flexor and dorsiflexor muscles, participants may increase stride width (SW) to enhance balance control without adjusting stride length (SL) or stride duration (SDN). This adaptive response was also observed after muscle fatigue of the knee extensors and flexors, where contributions from hip muscles during walking increased [25]. Through fatigue of the plantar flexor and dorsiflexor muscles, the study found that the reflex responses of the tibialis anterior and soleus muscles were significantly reduced, which may lead to a decrease in joint stiffness and stability [52,53]. Increased ankle plantar flexion should result in a decreased foot–floor angle [13]. Therefore, increased fatigue of the plantar flexor muscles could be a postural control attempt to reduce the likelihood of fall risk, possibly due to tibialis anterior fatigue, which could limit ankle dorsiflexion [54]. However, evidence supporting the relationship between the increased ankle plantar flexion angle and postural control mechanisms was lacking, and further investigation was needed to clarify or validate this hypothesis [13]. In general, walking, as a low- to moderate-intensity automated movement, may alleviate the effects of fatigue through compensatory mechanisms in the lower limb muscles for healthy adults, thereby demonstrating a reduction in fatigue.

Muscle fatigue coincided with compensatory gait changes that enhance stability, such as increased stride length (SL) [55], although this contrasts with some of our findings. Muscle fatigue may impair motor control by increasing force variability and limiting fast corrective torque generation [56,57]. Increasing stride width (SW) may provide a more significant margin for the centre of mass control. In the case of muscle fatigue of the knee extensors and flexors [58], participants reduced walking time, which also aids in balance control in the ML (medial–lateral) direction [59].

Recently, the study of human gait has become a major focus within the field of human motor control. Gait analysis could serve as a predictor of specific pathologies or help identify walking and posture issues, load abnormalities, and muscle failures that may not be detectable through standard clinical exams [60]. A key and direct step for future research is determining the critical threshold of fatigue, specifically, what level of fatigue significantly affects gait and increases the risk of falls or slips. When selecting fatigue protocols, it should be more aligned with daily life, favouring low-intensity and repetitive fatigue scenarios that require further investigation. Additionally, since falling while navigating stairs is common in everyday life, future studies should comprehensively examine stair gait following fatigue. We also recommend including the Center of Pressure (COP) as an important parameter in future studies. COP played a significant role in predicting fall rise, as it provided a comprehensive assessment of an individual’s balance and gait control under fatigue conditions [61,62]. Incorporating COP measurements can enhance our understanding of how fatigue affects performance and fall risk, offering valuable insights for both clinical and athletic settings.

### 4.3. Limitations

First, despite the extensive search, the number of studies included in the search was quite small. In addition, this review only included young adults as study participants, so the generalizability of the findings to other age groups may be limited. The results were primarily applicable to young adults and may not be directly extended to other age populations.

## 5. Conclusions

Through a systematic review analysis, we found that there was currently limited research on the effects of lower limb fatigue on gait, and there are controversies regarding certain indicators. In summary, lower limb muscle fatigue appears to affect the spatial–temporal characteristics of gait and has implications for joint kinematics and dynamics. However, the limited evidence and methodological differences have impacted the generalizability of the results. In contrast, due to compensatory mechanisms in the body after fatigue, new walking strategies may emerge, or the muscle strength required for walking may be lower, suggesting that fatigue might have a reduced impact on dynamic balance without significantly increasing the risk of injuries. Research on these indicators is essential for controlling balance and preventing injuries. Studies that want to use muscle fatigue more to model muscle injuries need to choose appropriate fatigue protocols, such as moderate to severe fatigue interventions using isokinetic muscle testers, to be able to model muscle injuries.

## Figures and Tables

**Table 1:** PICOS for systematic review study design.

PICOS	Description
Participant	The study will include healthy adults aged between 18 and 59. Participants should have no history of lower extremity diseases, disorders, or injuries that could affect their mobility or physical function.
Intervention	The non-invasive protocols for inducing lower limb muscle fatigue in a laboratory environment, such as isometric contractions, dynamic exercises, or repetitive tasks, allow for controlled and reproducible investigation of fatigue-related effects.
Comparison	Comparison of gait characteristics before and after fatigue.
Outcome	Lower limb joint biomechanical characteristics and balance indicators.
Study Design	Original intervention studies, including non-randomised controlled trials (non-RCTs).

**Table 2:** Search Strategy for Systematic Review.

Database	Search Strategy
CINAHL	((MH “Gait” OR “Gait Analysis”) AND (MH “Fatigue” OR “Muscle Fatigue”)) AND ((MH “Gait” OR “Walking Pattern”) AND (MH “Fatigue” OR “Muscle Fatigue” OR “Fatigue Assessment”))
Pubmed	(“gait” OR “gait analysis”) AND (“fatigue” OR “muscle fatigue”) AND (“gait” OR “gait analysis” OR “walking pattern”) AND (“fatigue” OR “muscle fatigue” OR “fatigue assessment”)
Web of Science	((gait OR “gait analysis”) AND (fatigue OR “muscle fatigue”)) and ((gait OR “gait analysis” OR “walking pattern”) AND (fatigue OR “muscle fatigue” OR “fatigue assessment”))
Cochrane	(“gait” OR “gait analysis”) AND (“fatigue” OR “muscle fatigue”) AND (“gait” OR “gait analysis” OR “walking pattern”) AND (“fatigue” OR “muscle fatigue” OR “fatigue assessment”)
Medline	(“gait” [Mesh] OR “gait analysis” [Mesh] OR “gait” OR “walking pattern”) AND (“fatigue” [Mesh] OR “muscle fatigue” [Mesh] OR “fatigue” OR “fatigue assessment”)

**Table 6:** Experimental setup and biomechanical methods.

	Michael A. Hunt., 2017 [16]	Fui Ling Lew., 2014 [13]	Heather S. Longpré., 2012 [22]	G. H. Murdock., 2011 [23]	Urs Granacher., 2010a [31]	Urs Granacher., 2010b [24]	Prakriti Parijat., 2008 [14]	Dennis Hamacher., 2016 [32]	F.A. Barbieri., 2014 [25]	Fabio Augusto Barbieri., 2013 [33]	Xingda Qu., 2011 [34]
Experimental setup	A walkway (10 m)	A walkway (12 × 1.5 m)	NE	A walkway (6 m)	A walkway (10 m)	NE	A walkway (15.5 × 1.5 m)	NE	10 cm height platform	A walkway (8 m)	NE
Biomechanical methods	Kinematics, kinetics and sEMG	Kinematics and kinetics	Kinematics, kinetics and sEMG	Kinematics, kinetics and sEMG	Kinetics	Kinematics and sEMG	Kinematics and kinetics	Inertial sensor	Kinematics and kinetics	Kinematics and kinetics	Kinematics

Surface Electromyography (sEMG); NE: The study does not explain it carefully.

**Table 7:** Effects of fatigue on gait indicators.

Study	Joint Moment	Joint Angle	sEMG	Spatiotemporal Parameters	Other Parameters
**Michael A. Hunt., 2017 [16]**	Knee extension 一 Knee flexion ↓ Plantar flexion ↓	Knee flexion ↑	MG, LG ↓	GV 一 SF ↑	
**Fui Ling Lew., 2014 [13]**		Knee 一 Plantar flexion ↑			SD ↑ RCOF 一
**Heather S. Longpré., 2012 [22]**	Knee extension ↓ Knee flexion 一	Knee 一	BF 一, RF ↓	GV 一 SL 一	
**G. H. Murdock., 2011 [23]**	Knee flexion ↓ Knee external Rotation ↓	Knee Adduction ↑ Knee 一	VM, RF ↓		
**Urs Granacher., 2010a [31]**				GV ↓ SL ↓ SLV 一	
**Urs Granacher., 2010b [24]**			TA ↓, SO 一		Angular velocity (plantar–dorsiflexion) ↑
**Prakriti Parijat., 2008 [14]**	Knee extension ↓	Knee flexion ↑ Plantar flexion ↑		GV ↓	RCOF ↑ HCV ↑
**Dennis Hamacher., 2016 [32]**					LDS ↑
**F.A. Barbieri., 2014 [25]**				SW ↑ STL 一 SDN 一	
**Fabio Augusto Barbieri., 2013 [33]**				GV ↑ SW ↑ SL ↑ SDN ↓	
**Xingda Qu., 2011 [34]**		Trunk ROM ↑, Hip ROM ↑, Knee ROM ↑		SW ↑ SL 一 SLV ↑	

All articles are in the fatigue group versus the no fatigue group. All of them are the fatigue side or the dominant side. ↓: decreased; ↑: increased; 一: no change; Medial Gastrocnemius (MG); Lateral Gastrocnemius (LG); Biceps Femoris (BF); Rectus Femoris (RF); Vastus Medialis (VM); Tibialis anterior (TA); Soleus (SO);Stride length variability (SLV); Gait velocity (GV); Stride frequency (SF); Stride length (SL); Step length (STL); Stride width (SW); Stride Duration (SDN); Local dynamic stability (LDS); Range of Motion (ROM); Heel Contact Velocity (HCV); Slip Distance (SD); Required coefficient of friction (RCOF).

## Data Availability

All data collected have been displayed in the publication.

## Supplementary Materials

The following supporting information can be downloaded at https://www.mdpi.com/article/10.3390/bioengineering12030225/s1, PRISMA checklist.
